# Patterns of cigarette, heated tobacco product, and nicotine vaping product use among Korean adults: Findings from the 2020 ITC Korea Survey

**DOI:** 10.18332/tid/186273

**Published:** 2024-04-18

**Authors:** Sungkyu Lee, Steve S. Xu, Mi Yan, Shannon Gravely, Anne C. K. Quah, Hong Gwan Seo, Sujin Lim, Sung-il Cho, Yeol Kim, Geoffrey T. Fong

**Affiliations:** 1 Korea Center for Tobacco Control Research and Education, Seoul, Republic of Korea; 2 Department of Psychology, University of Waterloo, Waterloo, Canada; 3 Korea National Cancer Center, Goyang, Republic of Korea; 4 National Tobacco Control Center, Korea Health Promotion Institute, Seoul, Republic of Korea; 5 Department of Public Health Science, Graduate School of Public Health, Seoul National University, Seoul, Republic of Korea; 6 School of Public Health Sciences, University of Waterloo, Waterloo, Canada; 7 Ontario Institute for Cancer Research, Toronto, Canada

**Keywords:** non-combustible nicotine products, heated tobacco products, nicotine vaping products, cigarettes, South Korea

## Abstract

**INTRODUCTION:**

Non-combustible nicotine products (NCNPs), such as heated tobacco products (HTPs) and nicotine vaping products (NVPs) have gained a significant nicotine market share in South Korea. This descriptive study examined patterns of regular cigarette and NCNP use among South Korean adults.

**METHODS:**

Data were from the 2020 International Tobacco Control Korea Survey and included 4016 adults (aged ≥19 years) in the Republic of Korea who were regularly (at least weekly) using at least one NCNP (NVP/HTP, n=2117) and/or smoked cigarettes (n=3763) at the time of the survey. Weighted descriptive estimates were computed to assess respondents’ nicotine product use among all respondents (exclusive, dual, or triple use). Thereafter, we identified sociodemographic characteristics associated with NCNP use (n=2117).

**RESULTS:**

Among Korean adults who were smoking cigarettes, 83.1% (95% CI: 81.6–84.6) did so exclusively, and 16.9% (95% CI: 15.4–18.4) smoked cigarettes and used NCNPs. Among those who used HTPs (n=1877), 14.9% (95% CI: 11.5–18.4) did so exclusively, 59.6% used HTPs and smoked cigarettes (95% CI: 55.4–63.1), 4.2% used HTPs and vaped (95% CI: 11.5–18.4), and 21.6% (95% CI: 18.9–24.2) used all three products. Of adults who used HTPs and smoked cigarettes, 86.6% smoked daily. Among those who vaped (n=865), 13.3% did so exclusively (95% CI: 9.4–17.1), 55.6% (95% CI: 49.6–61.5) vaped and smoked cigarettes, 5.1% (95% CI: 1.7–8.6) used HTPs and vaped, and 26.1% (95% CI: 22.1–30.1) used all three products. Of adults who vaped and smoked cigarettes, 82.4% (95% CI: 77.1–87.7) smoked daily.

**CONCLUSIONS:**

Cigarettes remain the most commonly used nicotine product in South Korea, and among adults using heated tobacco and/or vaping products, the majority were also smoking. Research is urgently needed to assess whether adults who are using an NCNP are doing so to quit, or rather to complement their cigarette smoking.

## INTRODUCTION

The global decline in cigarette sales over the past decade has prompted major tobacco companies, including Philip Morris International (PMI), British American Tobacco (BAT), Japan Tobacco International (JTI), and Korea Tobacco and Ginseng (KT&G), to change their tobacco business model to promote non-combustible nicotine products (NCNPs), such as heated tobacco products (HTPs) and nicotine vaping products (NVPs)^1^. The Republic of Korea (South Korea) was a key market for testing the viability of NCNPs for several reasons: 1) NCNPs can be legally sold; 2) cigarette smoking prevalence is extremely high among males, indicating a large existing market and smoking prevalence is low among females, indicating a potential market^2^; and 3) South Korea is a technological-driven society with a strong focus on adopting the latest technology^3^.

In South Korea, NVPs (‘cigalikes’ and refillable tank systems) first appeared in 2007 and were advertised as a smoking cessation aid or as a potential alternative to cigarettes^4^. Since then, the country has experienced cycles of rising and falling NVP use, beginning with the early devices that resembled traditional tobacco cigarettes (e.g. ‘cigalike’ devices) and refillable tank systems^5^. By May 2019, closed vaping systems (disposables and replaceable cartridges) had gained popularity. In 2020, the prevalence of vaping (at least monthly) among Korean adults was 3.2% (males 5.2%, females 1.1%)^5^.

PMI launched an HTP called IQOS in June 2017 as a logical response to Korea’s long history of NVPs and high smoking prevalence^6^. Following the launch of IQOS, BAT launched glo in 2017 and KT&G launched ‘lil’ in 2018^7,8^. To attract Koreans to HTPs, tobacco companies conducted extensive promotion strategies and used slogans like ‘95% less harmful’, ‘clean’, ‘free of fire, ash, and smoke’, and ‘free from cigarette-like smells’^7,8^. HTPs sales increased rapidly and accounted for 10.6% of total tobacco sales by 2020^9^. In 2020, 5.1% of Korean adults reported using HTPs at least monthly (8.4% of men, 1.5% of women). It is estimated that more than 90% of Koreans who used HTPs also smoked cigarettes and/or vaped^10^.

NCNPs account for a significant share of the South Korean nicotine market; however, little is known about patterns of use and sociodemographic characteristics of Korean adults who use NCNPs, including beyond an experimental basis. To our knowledge, this is the first study to report on population-level characteristics of adults in South Korea who smoke cigarettes, and who do or do not regularly use NCNPs. Using data from the International Tobacco Control (ITC) Korea 2020 Survey, we examined: 1) the proportion of Koreans who smoked cigarettes, vaped, and/or used HTPs, and who were using them exclusively or in combination (dual or triple use); 2) sociodemographic characteristics associated with NCNP use; 3) the frequency of cigarette smoking among those who vaped and/or used HTPs; and 4) device preferences among adults who use HTP and/or NVPs.

## METHODS

Data for this cross-sectional study came from the 2020 (Wave 1) ITC Korea Survey, which is a web-based survey of a national sample of 4740 adults (aged ≥19 years; 19 is the minimum legal age for purchasing cigarettes or NCNPs) who were recruited from Rakuten Insight’s web panel. Respondents are eligible for study recruitment if they: 1) smoke cigarettes at least weekly; 2) use HTPs and/or NVPs at least weekly; 3) quit smoking in the last 2 years and use HTPs and/or vape at least weekly; and 4) did not use any products at all (and either formally smoked or never smoked). These definitions allow us to differentiate people who use these products regularly rather than experimentally. Having this definition can also help us to differentiate exclusive use from co-use, which is popular in the Republic of Korea.

The online survey was conducted from 18 to 28 June 2020. All respondents provided consent prior to completing the survey. The response rate was 15.2%, and the cooperation rate was 97.4%. The sampling design, sample, and methods are described elsewhere^11,12^. Study procedures and materials were reviewed and cleared by the Research Ethics Board, University of Waterloo, Canada (REB#41512) and the Institutional Review Board of Korea Health Promotion Institute (#120160811107AN01-2004-HR-042-02).

The current study sample included Korean adults who regularly (at least weekly) smoked cigarettes, vaped and/or used HTPs, either exclusively or in combination: 3763 smoked cigarettes, 2117 used NCNPs (134 used HTPs, 65 vaped, 1123 used HTPs and smoked, 227 vaped and smoked, 55 used HTPs and vaped, and 514 used all three products). The study selection process is presented in Supplemental Figure 1.

### Measures

*Sociodemographic variables (independent variables)*

Sociodemographic variables included in the analyses were: age group (19–29, 30–39, 40–59, and ≥60 years), sex (male, female), annual household income [Korean Won was defined as: low: <30 million KRW (<22689 US$), moderate: 30 to <75 million KRW (22689 to 56724 US$), high: ≥75 million KRW (>56724 US$), or not reported], and education level (low: never attended school or dropped out of school/primary/middle school; moderate: completed high school/some university, no degree; high: completed university degree/postgraduate; or not reported).

*Status of HTP, NVP, and cigarette use (dependent variables)*

This study included those who regularly used NCNPs and/or cigarettes. To assess cigarette smoking status, the following question was asked: ‘How often do you currently smoke cigarettes?’. Those who answered ‘daily’ or ‘less than daily, but at least once a week’ were considered people who smoked cigarettes. HTP use status was assessed by: ‘How often do you currently use heated tobacco products?’. Those who answered ‘daily’ or ‘less than daily, but at least once a week’ were considered people who used HTPs. Vaping status was assessed by the following question: ‘How often do you currently use liquid e-cigarettes?’. Those who answered ‘daily’ or ‘less than daily, but at least once a week’ were considered people who vaped. Those who reported less than weekly use or not at all were excluded from the analyses.

*HTP or NVP device preferences*

Preference of HTP device was assessed using the following question: ‘Thinking now about the actual heating device that is used with tobacco sticks or capsules. Which of the following devices have you ever used?’. The response options included ‘IQOS’, ‘glo (mini, pro, or nano)’, ‘glo sens’, ‘Ploom TECH’, ‘Ploom TECH +’, ‘Ploom S’, ‘lil (plus, mini, or hybrid)’, and ‘other heated devices (not listed)’. Among people who had ever used HTPs at least weekly and reported using more than one HTP (brand) device, an additional question was asked: ‘Which heating device do you use most?’ with the same response options provided.

Among people who vaped at least weekly, their device preference was assessed using the following question: ‘Which of the following best describes the type of liquid e-cigarette you currently use most?’. Response options included ‘disposable, not refillable’, ‘uses replaceable pre-filled cartridges or pods’, and ‘has a tank that you fill with liquids’.

### Statistical analysis

Sample characteristics were examined using frequencies and unweighted percentages. All other analyses were conducted on weighted data. Survey weights were adjusted for unequal selection probabilities and designed to create a sample that is representative of the adult South Korean population. Survey weights were calibrated on their nicotine product use status, geographical region, and demographic measures to adjust for potential disproportional sampling of sub-groups (e.g. oversampling of respondents who used two products). The weight calibration was done using benchmarks from the 2019 Korea Community Health Survey. Details about weighting can be found elsewhere^11,12^.

Population descriptive characteristics were estimated using SURVEYFREQ, accounting for stratification and the corresponding weights of the types of nicotine products used. First, we assessed patterns of cigarette and NCNP use among all respondents (exclusive use, dual use, or triple products use, N=4016). Second, we assessed sociodemographic characteristics among those who reported using NCNPs (n=2117). Rao-Scott chi-squared tests were performed to compare differences between the four user-type groups (exclusive use, dual HTP-cigarette use, dual HTP-NVP use, and triple use) by sex, age group, education level, household income level, and cigarette smoking status (daily vs weekly). Statistical significance and confidence intervals were computed at the 95% confidence level. All analyses were conducted in SAS Version 9.4 (Version 9.4, SAS Institute Inc., Cary, NC, USA).

## RESULTS

Respondents’ baseline characteristics are presented in Table 1. The majority of Koreans who smoked cigarettes, used HTPs, and/or vaped, were male, from moderate-income households, and highly educated.

**Table 1:** Characteristics of the study samples: all who used at least one type of nicotine product (N=4016), who smoked cigarettes (N=3763), used heated tobacco products (N=1826) or vaped (N=860) at least weekly in the Republic of Korea, June 2020 (unweighted)

*Characteristics*	*All n (%)*	*Cigarette n (%)*	*HTP n (%)*	*NVP n (%)*
**Sex**				
Male	3181 (79.2)	3002 (79.8)	1373 (75.2)	607 (70.6)
Female	835 (20.8)	761 (20.2)	453 (24.8)	253 (29.4)
**Age** (years)				
19–29	566 (14.1)	511 (13.6)	270 (14.8)	192 (22.3)
30–39	1112 (27.7)	1028 (27.3)	600 (32.9)	299 (34.8)
40–59	1994 (49.7)	1889 (50.2)	842 (46.1)	339 (39.4)
≥60	344 (8.6)	335 (8.9)	114 (6.2)	30 (3.5)
**Annual household income**				
Low	551 (13.7)	520 (13.8)	155 (8.5)	106 (12.3)
Moderate	2307 (57.5)	2155 (57.3)	1033 (56.6)	485 (56.3)
High	1708 (26.8)	1015 (27.0)	615 (33.7)	257 (29.9)
Refused/don’t know	80 (2.0)	73 (1.9)	23 (1.3)	12 (1.4)
**Education level**				
Low	37 (0.9)	32 (0.9)	14 (0.8)	7 (0.8)
Moderate	773 (19.3)	722 (19.3)	239 (13.1)	153 (17.8)
High	3192 (79.8)	2997 (79.9)	1565 (85.7)	697 (81.0)

Data are unweighted and unadjusted. All: those who used at least one type of nicotine product at least weekly, including exclusive, dual and triple use. Cigarette: those who smoked cigarettes at least weekly (and may or may not use other products at least weekly). HTP: those who used HTPs at least weekly (and may or may not use other products at least weekly). NVP: those who vaped at least weekly (and may or may not use other products at least weekly).

### Proportions of Koreans who used any nicotine product, either exclusively or in combination

**Figure 1:**
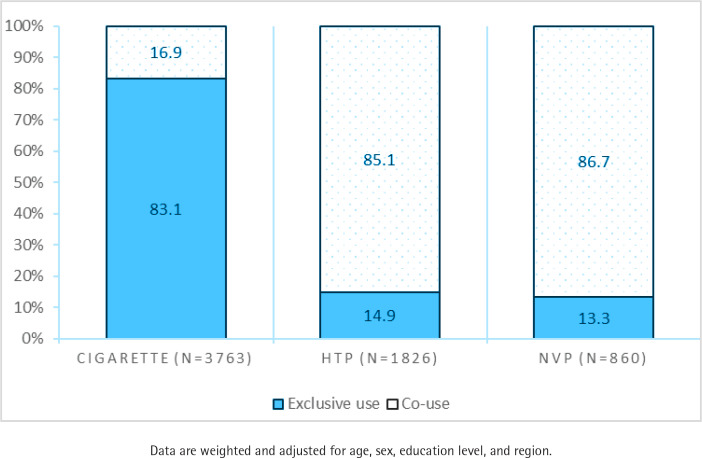
The proportion of South Korean adults who smoked cigarettes (N=3763), used heated tobacco products (N=1826), and/or vaped (N=860) at least weekly, either exclusively or in combination, in the Republic of Korea, June 2020

Figure 1 shows the weighted proportions of Koreans using any nicotine product, either exclusively or in combination. Among those who smoked cigarettes (n=3763), 83.1% (95% CI: 81.6–84.6) smoked cigarettes exclusively and 16.9% (95% CI: 15.4–18.4) smoked and used at least one NCNP (HTPs and cigarette, NVPs and cigarettes, or all three products). Of those who smoked cigarettes exclusively, 92.3% (95% CI: 90.7–93.9) smoked daily, and 7.7% (95% CI: 6.1–9.3) smoked weekly.

Among those who used HTPs (n=1826), 14.9% (95% CI: 11.5–18.4) used HTPs exclusively; 85.1% (95% CI: 81.6–88.5) used an HTP with at least one other NCNP (HTPs and cigarettes, HTPs and NVPs, or all three products), and 80.8% (95% CI: 76.7–84.9) smoked cigarettes and used HTPs. Of those who used HTPs and smoked, 86.6% (95% CI: 84.2–89.0) smoked daily, and 13.4% (95% CI: 11.0–15.8) smoked weekly.

Among those who vaped (n=860), 13.3% (95% CI: 9.4–17.1) vaped exclusively; 86.7% (95% CI: 82.9–90.6) vaped with at least one NCNP (NVPs and cigarettes, HTPs and NVPs, or triple products), and 81.6% (95% CI: 76.7–86.5) vaped and smoked. Of those who vaped and smoked, 82.4% (95% CI: 77.1–87.7) smoked daily and 17.6% (95% CI: 12.3–22.9) smoked weekly.

### Percentages and characteristics of Koreans who used HTPs

Table 2 presents the findings of the four groups who used HTPs in 2020: 14.9% exclusively, 59.6% used HTPs and smoked, 4.2% used HTPs and vaped, and 21.6% used all three products. Among all those who either used HTPs exclusively or used them with another nicotine product (cigarette or NVP), most were male, used HTPs daily, were from moderate-income households, highly educated, and aged 40–59 years. Most Koreans who used HTPs exclusively were male, used HTPs daily, were aged 40–59 years, from moderate-income households, and highly educated. The same characteristics were found among those who used HTPs exclusively or in combination with cigarettes and/or NVPs.

**Table 2:** The prevalence of HTP use and the characteristics of South Korean adults who used HTPs at least weekly in the Republic of Korea, June 2020 (N=1826) (weighted)

	*Any HTP*	*Exclusive HTP*	*Dual HTP-cigarette*	*Dual HTP-NVP*	*Triple*	*p*
**Overall**, n	1826	134	1123	55	514	
**Weighted % (95% CI)**	100	14.9 (11.5–18.3)	59.6 (55.4–63.1)	4.2 (1.4–7.1)	21.6 (18.9–24.2)	
***Characteristics***	***n (%) (95% CI)***	***n (%) (95% CI)***	***n (%) (95% CI)***	***n (%) (95% CI)***	***n (%) (95% CI)***	
**Sex**						
Male	1373 (92.3) (91.0–93.7)	107 (95.2) (92.4–97.9)	875 (92.5) (91.1–94.0)	30 (86.2) (71.9–100)	361 (91.0) (87.6–94.4)	<0.001
Female	453 (7.7) (6.3–9.0)	27 (4.8) (2.1–7.6)	248 (7.5) (6.0–8.9)	25 (13.8) (0.0–28.1)	153 (9.0) (5.6–12.4)	
**Age** (years)						
19–29	270 (19.7) (16.5–23)	24 (12.5) (4.1–21.0)	128 (18.4) (14.7–22.1)	11 (31.7) (0.0–66.9)	107 (26.0) (19.9–32.0)	<0.001 except Dual
30–39	600 (33.1) (29.8–36.5)	46 (29.3) (16.3–38.5)	349 (35.0) (38.9–47.2)	20 (18.1) (3.5–32.7)	185 (33.6) (27.7–39.5)	HTP-NVP <0.05
40–59	842 (43.5) (39.1–47.2)	58 (52.4) (39.5–65.4)	563 (43.1) (44.6–53.0)	22 (49.8) (15.0–84.5)	199 (37.2) (30.9–43.4)	
≥60	114 (3.7) (2.3–5.1)	6 (5.7) (0.0–12.6)	83 (3.5) (2.2–4.7)	2 (0.4) (0.0–3.1)	23 (3.3) (0.8–5.8)	
**Annual household income**						
Low	155 (12.8) (10.2–15.3)	14 (8.4) (2.4–14.4)	88 (12.6) (9.3–15.8)	4 (3.2) (0.0–7.5)	49 (18.3) (12.6–24.0)	<0.001
Moderate	1033 (56.7) (52.9–60.4)	77 (58.5) (45.7–71.2)	638 (57.1) (52.9–61.4)	38 (66.0) (31.3–100.0)	280 (52.3) (45.8–58.8)	
High	615 (29.4) (26.0–32.9)	38 (30.8) (18.5–43.2)	384 (29.3) (25.6–33.0)	12 (30.7) (0.0–60.7)	181 (28.7) (23.0–34.3)	
Refused/don’t know	23 (1.1) (0.6–1.7)	5 (2.3) (0.3–4.5)	13 (1.0) (0.4–1.6)	1 (0.2) (0.0–0.7)	4 (0.8) (0.0–1.9)	
**Education level**						
Low	14 (1.3) (0.5–2.1)	3 (1.8) (0.0–4.0)	6 (0.9) (0.0–1.7)	1 (1.4) (0.0–4.1)	4 (2.2) (0.0–4.6)	<0.001
Moderate	239 (32.0) (28.3–35.8)	21 (17.4) (8.7–26.1)	138 (34.8) (30.1–39.5)	9 (4.3) (0.0–9.1)	71 (40.0) (33.1–46.9)	
High	1565 (66.6) (62.9–70.4)	108 (80.8) (71.9–89.7)	975 (64.3) (59.6–69.1)	45 (94.4) (88.4–100)	437 (57.8) (51.0–64.6)	
**HTP use frequency**						
Daily	1175 (63.9) (60.3–67.5)	82 (58.9) (45.8–71.9)	744 (66.2) (62.2–70.3)	19 (64.4) (38.1–90.7)	330 (60.9) (54.5–67.3)	<0.01 except exclusive
Weekly	651 (36.0) (32.5–39.7)	52 (41.1) (25.3–51.0)	379 (33.8) (29.7–37.8)	36 (35.6) (9.3–61.8)	184 (39.1) (32.7–45.5)	HTP=0.18 Dual HTP-NVP=0.30

Data are weighted and unadjusted. Descriptive estimates were generated using SURVEYFREQ, accounting for stratification and the corresponding weights of the type of nicotine products(s) used. Rao-Scott chi-squared tests were performed to test differences between the three groups. Any HTP: all use HTPs at least weekly (and may or may not use other products at least weekly). Exclusive HTP: Only use HTPs at least weekly (do not use cigarettes or NVPs at least weekly). Dual HTP-cigarette: At least weekly use of both HTPs and cigarettes (do not vape at least weekly). Dual HTP-NVP: At least weekly use of both HTPs and NVPs (do not use cigarettes at least weekly). Triple: Use of all three products at least weekly.

### HTP device preference

**Figure 2:**
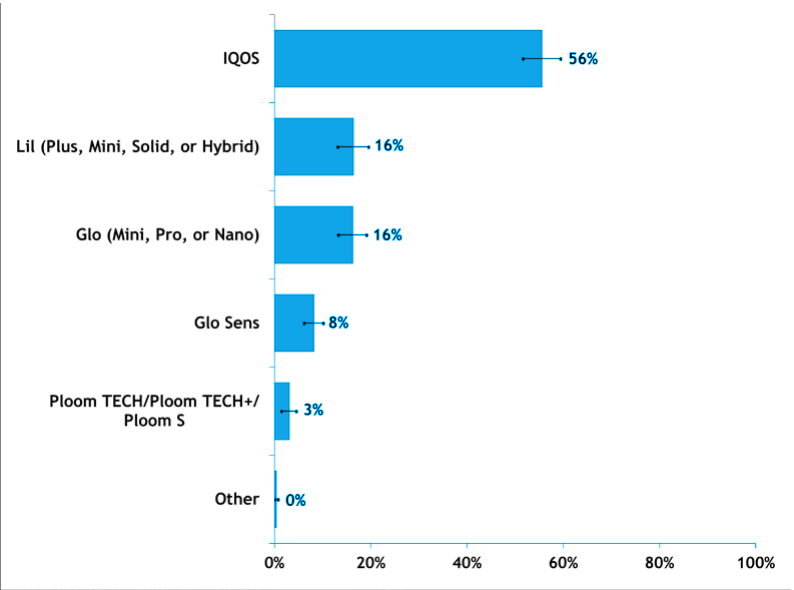
HTP device use preferences among South Korean adults who used HTPs at least weekly, in the Republic of Korea, June 2020 (N=1748)

Figure 2 presents device preferences (devices used most often) among adults who used HTPs. IQOS was the most popular HTP device (55.6%), followed by ‘lil (plus, mini, or hybrid)’ (16.4%), ‘glo (mini, pro, nano)’ (16.2%), and ‘glo sens’ (8.2%). The least popular HTP devices were JTI’s Ploom (TECH, TECH+, S) (3.1%).

### Proportions and characteristics of Koreans who vaped

Table 3 presents the findings of the four groups of Koreans who vaped in 2020: 13.3% vaped exclusively, 55.6% vaped and smoked, 5.1% vaped and used HTPs, and 26.1% used all three products. Among all who vaped, most were males, from moderate-income households, highly educated, vaping daily, and aged 19–29 years. The majority of Koreans who vaped exclusively were male, vaping daily, from moderate-income households, highly educated, and aged 40–59 years. The majority of Koreans who vaped and smoked were males, vaping daily, from moderate-income households, highly educated, and aged 19–29 years. The majority of Koreans who used HTPs and vaped, and those who used all three products were males, vaping weekly, from moderate-income households, highly educated, and aged 40–59 years.

**Table 3:** The prevalence of vaping and the characteristics of South Korean adults who vaped at least weekly in the Republic of Korea, June 2020 (N=860) (weighted)

	*Any NVP*	*Exclusive NVP*	*Dual NVP-cigarette*	*Dual HTP-NVP*	*Triple*	*p*
**Overall**, n	860	64	227	55	514	
**Weighted % (95% CI)**	100	13.3 (9.4–17.1)	55.6 (49.6–61.5)	5.1 (1.7–8.6)	26.1 (22.1–30.1)	
***Characteristics***	***n (%) (95% CI)***	***n (%) (95% CI)***	***n (%) (95% CI)***	***n (%) (95% CI)***	***n (%) (95% CI)***	
**Sex**						
Male	607 (89.7) (86.3–93.2)	42 (87.8) (81.9–93.8)	174 (89.9) (84.1–95.6)	30 (86.2) (71.9–100.0)	361 (91.0) (87.6–94.4)	<0.001
Female	253 (10.3) (6.8–13.7)	22 (12.2) (6.2–18.1)	53 (10.1) (4.4–15.9)	25 (13.8) (0.0–28.1)	153 (9.0) (5.6–12.4)	
**Age** (years)						
19–29	192 (36.2) (29.9–42.5)	20 (32.7) (18.1–47.3)	54 (42.2) (32.5–51.9)	11 (31.7) (0.0–66.9)	107 (26.0) (19.9–32.0)	<0.001 except Dual
30–39	299 (26.9) (21.8–32.0)	18 (26.0) (12.7–39.2))	76 (24.7) (16.8–32.7)	20 (18.1) (3.5–32.7)	185 (33.6) (27.7–39.5)	HTP-NVP <0.05
40–59	339 (35.3) (29.5–41.1)	25 (41.0) (27.7–57.8)	93 (31.8) (23.2–40.3)	22 (49.8) (15.0–84.5)	199 (37.2) (30.9–43.4)	
≥60	30 (1.6) (0.4–2.9)	1 (0.3) (0.0–0.9)	4 (1.3) (0.0–3.1)	2 (0.4) (0.0–3.1)	23 (3.3) (0.8–5.8)	
**Annual household income**						
Low	106 (19.9) (14.7–25.1)	13 (15.9) (5.4–26.5)	40 (23.2) (14.9–31.5)	4 (3.2) (0.0–7.5)	49 (18.3) (12.6–24.0)	<0.001
Moderate	485 (55.1) (48.8–61.3)	37 (62.6) (47.9–77.2)	130 (53.6) (43.9–63.2)	38 (66.0) (31.3–100)	280 (52.3) (45.8–58.8)	
High	257 (23.3) (18.0–28.6)	13 (19.4) (7.2–31.5)	51 (21.0) (13.0–29.0)	12 (30.7) (0.0–60.7)	181 (28.7) (23.0–34.3)	
Refused/Don’t know	12 (1.7) (0.0–3.4)	1 (2.1) (0.0–6.2)	6 (2.2) (0.0–5.1)	1 (0.2) (0.0–0.7)	4 (0.8) (0.0–1.9)	
**Education level**						
Low	7 (1.4) (0.0–3.0)	1 (0.6) (0.0–1.8)	1 (1.3) (0.0–3.8)	1 (1.4) (0.0–4.1)	4 (2.2) (0.0–4.6)	<0.001
Moderate	153 (44.7) (38.4–51.1)	21 (35.3) (20.8–49.8)	52 (53.0) (43.5–62.4)	9 (4.3) (0.0–9.1)	71 (40.0) (33.1–46.9)	
High	697 (53.8) (47.5–60.2)	42 (64.1) (49.6–78.6)	173 (45.7) (36.4–55.1)	45 (94.4) (88.4–100.0)	437 (57.8) (51.0–64.6)	
**Use frequency**						
Daily	406 (50.0) (43.8.3–56.3)	38 (70.8) (58.1–83.4)	111 (50.2) (40.5–59.8)	15 (12.4) (1.5–23.3)	242 (46.5) (40.0–52.9)	<0.01 except
Weekly	454 (50.0) (43.7–56.2)	26 (29.2) (16.4–41.9)	116 (49.8) (40.2–59.5)	40 (87.6) (76.7–98.5)	272 (53.5) (47.1–60.0)	Triple=0.28

Data are weighted and unadjusted. Rao-Scott chi-squared tests were performed to test differences between the three groups. All NVP: all vape at least weekly (and may or may not use other products at least weekly). Exclusive NVP: Only vape at least weekly (do not use HTPs or cigarettes at least weekly). Dual NVP-cigarette: Vape and smoke cigarettes at least weekly (do not use HTPs at least weekly). Dual HTP-NVP: Use HTPs and vape at least weekly (do not use cigarettes at least weekly). Triple: Use of all three products at least weekly.

### NVP device preference

**Figure 3:**
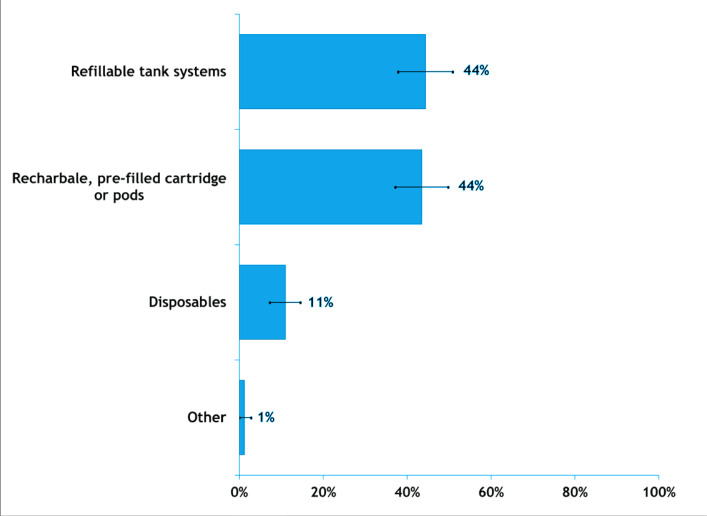
Vaping device use preference among South Korean adults who vaped at least weekly, in the Republic of Korea, June 2020 (N=805)

Figure 3 presents the weighted preference over NVP device types among adults who used NVPs. The refillable tank systems were the most popular (44.4%), followed by rechargeables (e.g. Juul and lil vapor) (43.5%), and disposables (e.g. Bubblemon, Monster vapor bar) (11.0%).

## DISCUSSION

This study described the use patterns of cigarettes and/or NCNPs among Korean adults in 2020. We also examined sociodemographic characteristics among those who used HTPs and vaped. We found that among Korean adults who smoked cigarettes, 83% smoked exclusively, and 17% smoked and used NCNPs (used HTPs and/or vaped). Among Koreans who used HTPs and smoked, 87% smoked daily. Among Koreans who vaped and smoked, 82% smoked daily. Among Koreans who used HTPs, the two most common HTP use patterns were dual use with smoking (60%), and triple use (22%). Because of the high rate of co-use and daily smoking habits, it appears that Koreans are using HTPs to complement rather than substitute cigarette smoking.

The findings of this study are consistent with those of other studies indicating that Koreans are using HTPs to complement cigarette smoking^10,13,14^. Moreover, the high rate of daily smoking (>90%) suggests longitudinal studies are needed to examine whether people are using HTPs to help to quit their cigarette smoking or merely out of convenience (e.g. at home or at work). HTP co-use patterns may have contributed to the minor reduction in cigarette sales and the rapid rise of HTP sales and prevalence, but not to the decline in smoking rates.

Among Koreans who used HTPs, most were male, used HTPs daily, aged 40–59 years, with a moderate income, and highly educated. Korean adults who used HTPs appear to share a similar sociodemographic profile as adults who used HTPs in Japan, with the exception that most Japanese were from high-income households. Compared to Japan, in 2018, a slightly higher proportion of Koreans who used HTPs were males (92% vs 76%) and smoked cigarettes (81% vs 68%)^15^. Another HTP study conducted in 28 European countries in 2020, indicated that most Europeans who used HTPs were mostly male, highly educated, and smoked cigarettes, consistent with our findings in South Korea. However, Europeans who used HTPs appear to be younger (aged ≤39 years) compared to those who used them in South Korea^16^.

Our study reveals that IQOS was the most popular HTP device in 2020. In many other countries, IQOS had a similar leading position, which likely contributed to PMI’s global dominance in HTPs^17^. As of December 2022, IQOS was available in 73 countries and was used by approximately 24.9 million adults worldwide. In 2022, IQOS sales accounted for 13.6% of its total tobacco sales, but approximately 32% of its total revenue^18^. Thus, IQOS does appear to be strongly contributing to PMIs financial portfolio.

The top two NVP use patterns among Koreans were dual use of HTPs and smoking (56%) and triple product use (26%). The high co-use patterns suggest Koreans also vaped as a complement to cigarette smoking and did not intend to completely replace cigarettes with NVPs. According to an early study conducted in South Korea, adults who smoked and vaped smoked more cigarettes and had higher levels of cotinine than those who only smoked cigarettes exclusively^19^. Both studies show that NVPs are commonly used as a complement to cigarette smoking.

Vaping by Korean females (any NVP type 10%; exclusive NVP 12%) is higher than that of HTPs (any NVP type 8%, exclusive NVP 5%). Traditionally, smoking among Korean women is culturally taboo and often associated with negative images, such as ‘unladylike’, ‘irresponsible’, or ‘rebellious’^20^. The lack of tobacco smell in NVPs may have attracted many Korean females to start and maintain vaping in order to avoid the stigma associated with female smoking^21^.

With regard to the popularity of NVP devices, refillable tank systems were the most popular (44%), followed by pre-filled cartridge devices (43%), and disposables (11%). A new generation of prefilled cartridge devices (e.g. lil vapor and Juul pod), which were introduced in 2019, became popular during our survey period. The popularity of pre-filled NVPs, however, did not last long for two reasons. First, the Korean government strongly warned the public to stop vaping in October 2019^22^. Second, as per the Korea Chemical Laws, individuals who wish to sell nicotine limit above 10 mg/mL must have chemical experts and proper facilities, which are difficult to provide. Consequently, the nicotine limit of most e-liquid imported into the Korea market was below 1%. However, there is no nicotine limit for cigarettes and HTPs. As a result, Juul Labs left the Korean market and KT&G stopped selling its ‘lil vapor’ after May 2021, which used to be the top two pre-filled cartridge system manufacturers^23^. Further research is needed to examine how the discontinuation of these prefilled cartridge devices on the open market has affected preferences and nicotine consumption habits in South Korea.

We found that about a fifth of those using HTPs were also vaping and smoking, and about a quarter of those vaping were also using HTPs and smoking. A 2018–2019 Italian study found that triple product use accounted for 40% among those who used HTPs and 15% among those who vaped^24^. According to a study conducted by Seo et al.^14^, there is no significant difference in motivations for initiating and regularly using HTPs (i.e. curiosity, reduced odor, perceived harm reduction compared to cigarettes) between individuals who used HTPs and smoked, and those who used all three products. Nevertheless, those who used all three products consistently expressed a wider variety of less frequently cited reasons for their use (e.g. enjoyment of flavor, attraction to heating/charging devices, attraction for HTP technology). Because most Koreans who vaped also smoked before the introduction of HTPs, it is likely that some Koreans who vaped and smoked, initiated HTPs use when HTPs became available^25^.

### Limitations

There are some limitations to this study that should be considered when interpreting the results: First, the use of HTPs and vaping in this study was self-reported. A wide range of overlapping characteristics and a lack of distinctive product design features may have resulted in measurement errors. For example, in the Korean language, NVPs are called ‘liquid e-cigarettes’, while HTPs are called ‘cigarette-type e-cigarettes’ or ‘solid e-cigarettes’, which may have caused some confusion among Koreans who use them^5^. Second, it is possible that some Korean respondents were excluded from the survey, for example, those who were not interested in taking part in online surveys, regardless of sampling and weighting designed to ensure national representativeness, thus generalizability to the overall Korean population who use nicotine should be interpreted with caution. Third, due to the cross-sectional design, our findings only reflect a snapshot of a rapidly changing nicotine market in the Republic of Korea. Fourth, this study was conducted in the Republic of Korea where the sale of HTPs and NVPs is legal, thus the findings may not be applicable to other countries where these products cannot be sold legally (e.g. in Japan where nicotine containing NVPs are not legal to be sold, but HTPs are).

## CONCLUSIONS

We found that most Koreans who used non-combustible nicotine products also smoked, and most of them smoked daily. At least 20% of Koreans used all three nicotine products in 2020. The majority of Koreans use NCNPs to complement rather than substitute cigarettes. As a result, the use of NCNPs did not decrease smoking prevalence or benefit public health.

## Supplementary Material



## Data Availability

In each country participating in the international Tobacco Control Policy Evaluation (ITC) Project, the data are jointly owned by the lead researcher(s) in that country and the ITC Project at the University of Waterloo. Data from the ITC Project are available to approved researchers 2 years after the date of issuance of cleaned data sets by the ITC Data Management Centre. Researchers interested in using ITC data are required to apply for approval by submitting an International Tobacco Control Data Repository (ITCDR) request application and subsequently to sign an ITCDR Data Usage Agreement. The criteria for data usage approval and the contents of the Data Usage Agreement are described online (http://www.itcproject.org).
