# Supplementary figures and images for: Patterns of cigarette, heated tobacco product, and nicotine vaping product use among Korean adults: Findings from the 2020 ITC Korea Survey
Source: Tob Induc Dis. 2024 Apr 18;22:10.18332/tid/186273. doi: 10.18332/tid/186273 (PMC11025448; doi:10.18332/tid/186273)

## Supplementary Figure

Supplemental Figure 1. Study Flow Diagram

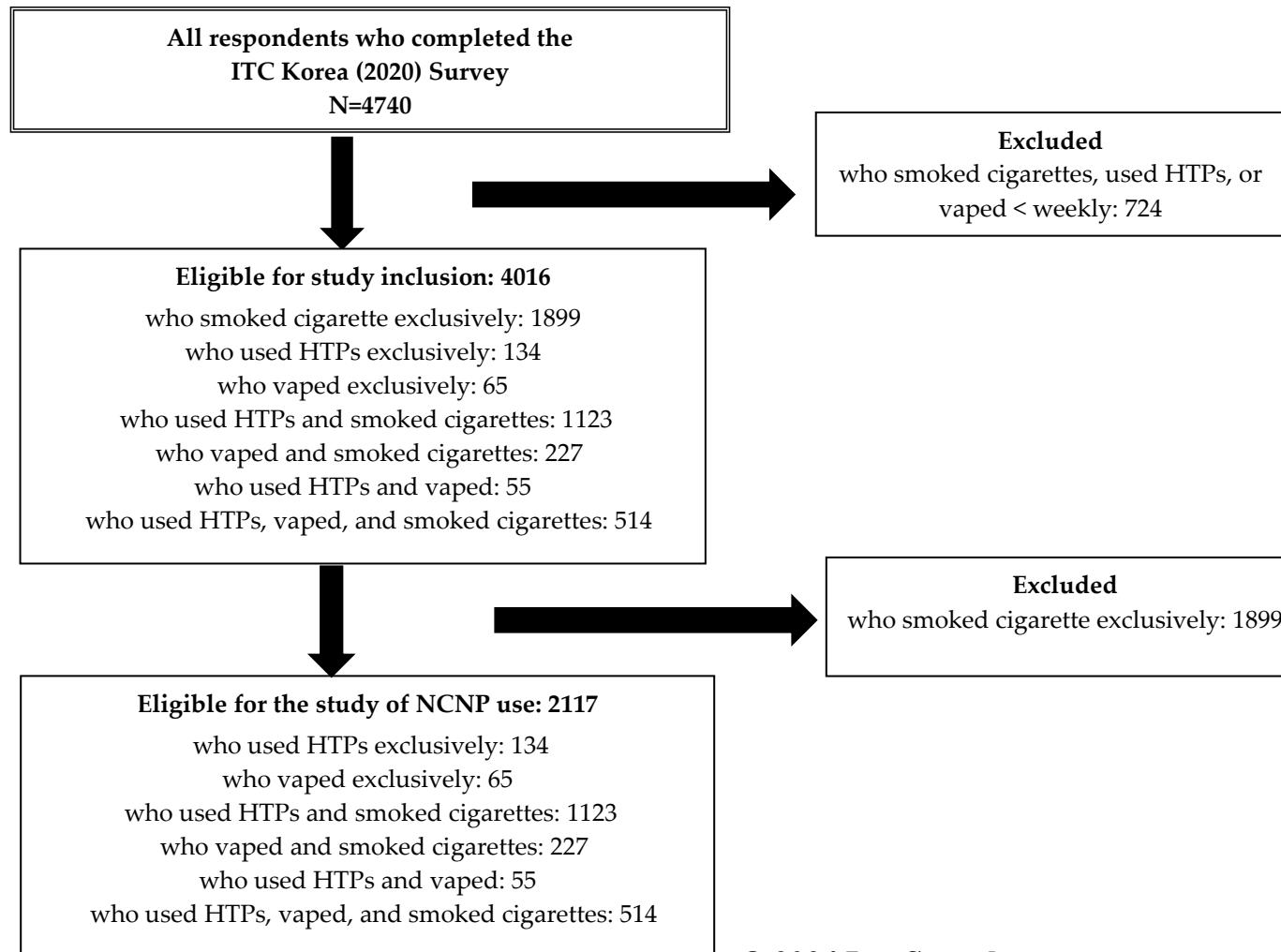

Supplement: Supplementary file 1 [file TID-22-63-s1.pdf]
